# Synergistic Inhibiting Effect of Phytochemicals in *Rheum palmatum* on Tyrosinase Based on Metabolomics and Isobologram Analyses

**DOI:** 10.3390/molecules28030944

**Published:** 2023-01-17

**Authors:** Yin Xiong, Hye Kyong Kim, Övgü Çelikler Özer, Bert van Duijn, Henrie A. A. J. Korthout, Lihong Zi, Ang Cai

**Affiliations:** 1 Fytagoras B.V., Sylviusweg 72, 2333 BE Leiden, The Netherlands; 2 Faculty of Life Science and Technology, Kunming University of Science and Technology, Kunming 650000, China; 3 Leiden University—European Center for Chinese Medicine and Natural Compounds, Institute of Biology, Leiden University, Sylviusweg 72, 2333 BE Leiden, The Netherlands; 4 Natural Product Laboratory, Institute of Biology, Leiden University, Sylviusweg 72, 2333 BE Leiden, The Netherlands; 5 The Scientific and Technological Research Council of Turkey, 06100 Ankara, Turkey; 6 Plant Biodynamics Laboratory, Institute of Biology, Leiden University, Sylviusweg 72, 2333 BE Leiden, The Netherlands; 7 Department of Biomedical and Pharmaceutical Sciences, College of Pharmacy, University of Rhode Island, Kingston, RI 02881, USA

**Keywords:** tyrosinase inhibitory activity, *Rheum palmatum*, metabolomics, isobologram analysis, orthogonal partial least square-modelling, catechin, gallic acid, synergistic effect

## Abstract

Tyrosinase (TYR) plays a key role in the enzymatic reaction that is responsible for a range of unwanted discoloration effects, such as food browning and skin hyperpigmentation. TYR inhibitors could, therefore, be candidates for skin care products that aim to repair pigmentation problems. In this study, we used a metabolomics approach combined with the isobologram analysis to identify anti-TYR compounds within natural resources, and evaluate their possible synergism with each other. *Rheum palmatum* was determined to be a model plant for observing the effect, of which seven extracts with diverse phytochemicals were prepared by way of pressurized solvent extraction. Each *Rheum palmatum* extract (RPE) was profiled using nuclear magnetic resonance spectroscopy and its activity of tyrosinase inhibition was evaluated. According to the orthogonal partial least square analysis used to correlate phytochemicals in RPE with the corresponding activity, the goodness of fit of the model (R_2_ = 0.838) and its predictive ability (Q_2_ = 0.711) were high. Gallic acid and catechin were identified as the active compounds most relevant to the anti-TYR effect of RPE. Subsequently, the activity of gallic acid and catechin were evaluated individually, and when combined in various ratios by using isobologram analysis. The results showed that gallic acid and catechin in the molar ratios of 9:5 and 9:1 exhibited a synergistic inhibition on TYR, with a combination index lower than 0.77, suggesting that certain combinations of these compounds may prove effective for use in cosmetic, pharmaceutical, and food industries.

## 1. Introduction

Melanin is the primary determinant of the skin color in humans, of which the overproduction may cause skin disorders such as hyperpigmentation and melanoma, and could be genotoxic as well [1]. Familiar agents such as hydroquinone and kojic acid (KA) are well known for their strong effects on preventing the formation of melanin, but are also responsible for unwanted side effects, or even skin toxicity [2]. Considering the increasing worries related to synthetic substances, the development of skin-care products from natural resources with a more secure and stable effect has become the current trend in the cosmetic industry [3].

In the synthesis of melanin, the multi-copper enzyme tyrosinase plays the key role in the first two rate-limiting steps [4]. It catalyzes the hydroxylation of L-tyrosine to 3,4-dihydroxyphenylalanine (L-DOPA), and subsequently the oxidation of L-DOPA to *o*-quinones. This highly conserved enzymatic reaction is responsible for several unwanted discoloration effects, such as food browning and skin hyperpigmentation. Therefore, tyrosinase (TYR) inhibitors could be the candidates for skin care products to repair pigmentation problems such as freckles, melasma, age spots, and acne scars. An interesting source of TYR inhibitors are plants rich in various active phytochemicals, of which the extracts have been used in cosmetic products to reduce hyperpigmentation disorders. The most commonly used method to identify anti-TYR components from plants is based on the reaction between TYR and its substrate of L-tyrosine or L-DOPA, followed by the fractionation using column chromatography and subsequent identification of anti-TYR components from the active fractions. However, this method is often time-consuming and sometimes fails to consider the synergism or antagonism among those compounds [5,6].

Plants from the Polygonaceae family that are rich in anti-TYR and antioxidant compounds have been used as a source of photo-protectants and skin-care products [7]. As a medicinal plant from Polygonaceae, *Rheum palmatum* (RP) has been cultivated and commercialized as ornamental rhubarb. On the other hand, this plant is a widely-used traditional medicinal herb for the treatment of constipation, jaundice, gastrointestinal hemorrhages, and ulcers [8]. The major constituents in RP, such as chrysophanol, physcion, emodin, aloe emodin, and rhein, all show interesting biological activities including laxative, anti-oxidant, and anti-inflammatory properties [9]. *Rheum rhaponticum* L., which belongs to the same genus as RP, showed strong anti-TYR activity and shared many common ingredients with RP [10]. However, the identification of TYR-inhibiting compounds from RP has not been described yet to our knowledge. Therefore, in this study, a nuclear magnetic resonance (NMR)-based metabolomics approach, together with multivariate analysis, was performed to identify anti-TYR ingredient(s) from RP. This approach has been shown to be efficient to find active compounds in the complex mixture of plant extracts, with the advantage of providing comprehensive qualitative and quantitative information in a time-saving way, compared with traditional strategies such as the bioassay-guided fractionation [11]. Furthermore, the synergistic effect of two identified components from RP was investigated, which has not been explored to our knowledge. We hope the methods and results of the study could support the potential applications of RP and its active compounds in medicinal, food, or cosmetic industries.

## 2. Results

### 2.1. Inhibiting Effect of RP Extracts (RPEs) on TYR

**Figure 1:**
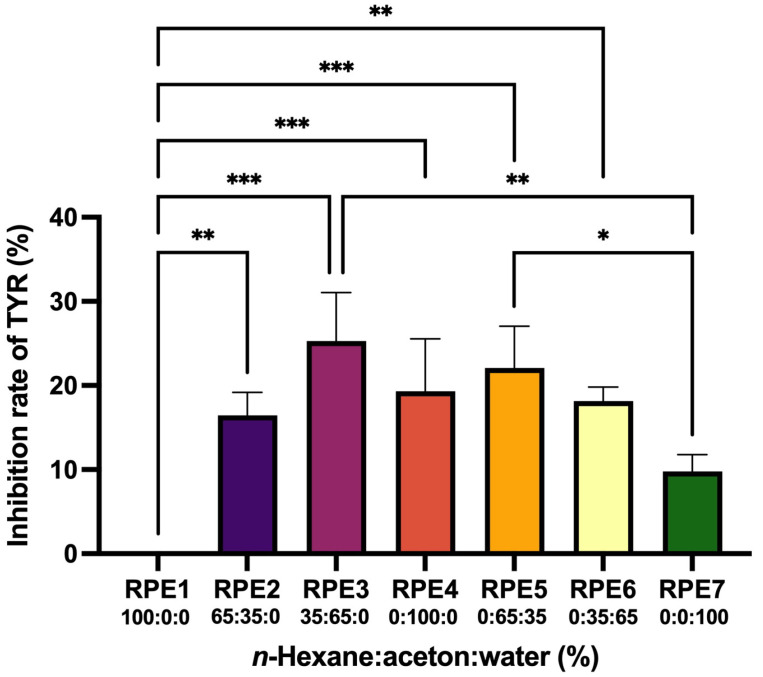
TYR-inhibition of different RPEs. RPE1 to RPE7 were extracts prepared by the mixture of *n*-hexane–acetone–water of different proportions. Inhibition of the positive control, KA at 0.94 μM, was 95.1 ± 0.4%. Mean value ± standard deviation was expressed (*n* = 3). RPE, *Rheum palmatum* extract; TYR, tyrosinase. * *p* < 0.05, ** *p* < 0.01, and *** *p* < 0.001.

Generally, a single solvent is used to obtain an initial plant extract in studies of natural products. However, it is sometimes impossible to know whether the most active compounds have been extracted from the plant by a single solvent. In this study, we used a mixture of solvents *n*-hexane–acetone–water to extract diverse phytochemicals from non-polar to polar by way of pressurized solvent extraction (PSE) [12]. This resulted in 21 different RPEs (seven compositions of *n*-hexane–acetone–water, triplicates for each composition). The TYR inhibitory effect of each RPE at a concentration of 200 μg/mL was shown in Figure 1. The TYR inhibition rate of the positive control of KA at 0.94 μM was 95.1 ± 0.4%. Most extracts showed a mild inhibitory activity ranging from 8% to 32%. Among these, the most active extract was RPE3 which was extracted with the solvent *n*-hexane–acetone–water at the ratio of 35:65:0, with the inhibition rate of 26 ± 3%.

### 2.2. Correlation of NMR Data and Activity Results

Different extracts prepared from PSE were analyzed by ^1^H-NMR spectroscopy. Since the solubilities of those samples were different, dimethyl sulphoxide (DMSO) was selected as a solvent for NMR analysis. Most extracts were well dissolved in DMSO-d_6_. Visual inspection of the ^1^H-NMR spectrum revealed that most of the aromatic compounds were extracted from RPE2 (n-hexane–acetone–water = 65:35:0) till RPE5 (n-hexane–acetone–water = 0:65:35). While in the RPE1 (n-hexane 100%) mostly lipid and fatty acids were present, and in the RPE7 (100% water) mostly sugars were extracted. From the aromatic area, the most prominent signals were coming from gallic acid (GA) and catechin derivatives. Typical ^1^H-NMR spectra of RPE were shown in Figure S1. In the case of anthraquinone compounds, the most well-known compounds in RP such as rhein (8.15, s, H-4) and chrysphanol (7.62, s, H-4; 7.22, s, H-2), signals could be recognized [13]. However, the signals were very low, and the rest of the signals were difficult to identify as they were in the congested area.

In order to examine the correlation between the TYR-inhibition rates of RPE samples and the corresponding phytochemicals within them, orthogonal partial least squares to latent structures (OPLS) modelling was applied. OPLS is a multivariate linear regression model that can discriminate between the bioactivity-related variables and non-related variables. In our study, OPLS was employed to determine the correlation of ^1^H-NMR signals (X variables) with the bioactivity (Y variables), which was the TYR-inhibition rate of each RPE at the concentration of 200 ug/mL. From the ^1^H-NMR and bioactivity data, an OPLS model with one predictive and two orthogonal components was obtained. The goodness of fit of the model (R_2_ = 0.838) and its predictive ability (Q_2_ = 0.711) were high. The OPLS model was validated further by CV-ANOVA and the model was significant with *p* < 0.05.

**Figure 2:**
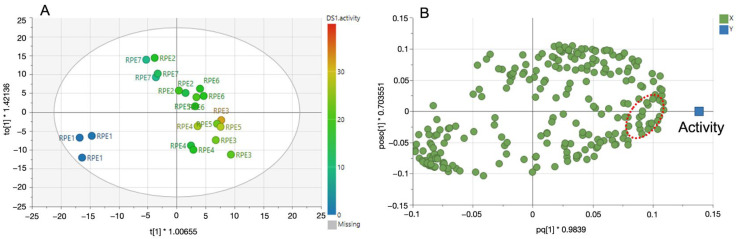
OPLS score plot (**A**) and loading plot (**B**) of RPEs. The scores are colored according to the TYR inhibition activity. (**A**): The label codes consist of RPE samples prepared by different extraction solvents, as described in Materials and Methods. (**B**): Loading plot presents the NMR signals (green dots) which contributed for the bioactivity (blue square). Green dots in the red circle refer to the characteristic proton NMR signals at δ 6.90 (s), δ 5.87 (d, J = 2.4 Hz), and δ 5.67 (d, J = 2.4 Hz). OPLS, orthogonal partial least squares; RPE, *Rheum palmatum* extract; TYR, tyrosinase.

**Figure 3:**
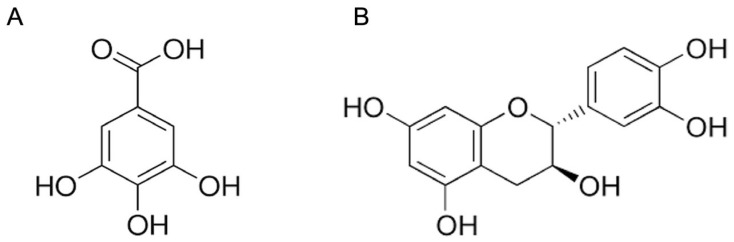
The chemical structures of (**A**) GA and (**B**) CH. GA, gallic acid; CH, catechin hydrate.

The score plot of the OPLS analysis showed that the activity was mostly correlated with the samples of RPE3 (65:35:0). Additionally, those samples were relatively well separated (Figure 2A). By examining the corresponding loading plot, the characteristic signals at δ 6.90 (s), δ 5.87 (d, J = 2.4 Hz), and δ 5.67 (d, J = 2.4 Hz) were identified to be correlated to the enhanced bioactivity very well (Figure 2B). Those signals were identified to be GA and catechin, respectively, by using an in-house database. As GA and catechin were the main compounds in the most active extracts and OPLS analysis indicated they were mainly responsible to the activity (Figure 2B), we then evaluated the TYR inhibitory activity of GA and catechin hydrate (CH) individually to confirm the prediction results. The structures of the two compounds were shown in Figure 3.

### 2.3. Inhibitory Effect of GA and CH on TYR

**Figure 4:**
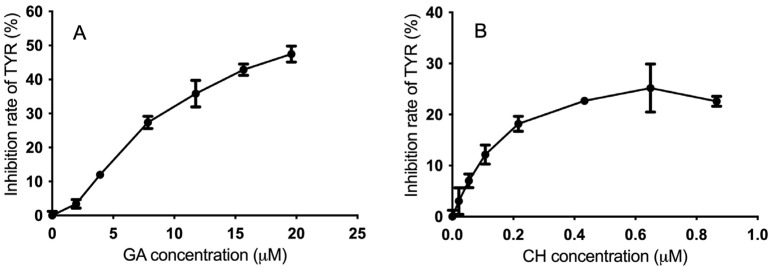
Concentration–response curves of (**A**) GA and (**B**) CH on TYR inhibition (*n* = 3). GA, gallic acid; CH, catechin hydrate; TYR, tyrosinase.

The concentration–response curves of TYR exposed to GA and CH individually were shown in Figure 4. According to Figure 4A, the inhibitory activity of GA on TYR was increased in a concentration-dependent manner, which was consistent with an earlier report [14]. The IC_50_ of GA was 20 µM, whereas CH could only inhibit TYR with a maximum inhibition rate of around 25% (Figure 4B).

From the results, the TYR inhibitory effect of individual GA or CH was not strong. Considering the common fact that the combination of two or more single compounds might produce a synergistic effect, the possible synergism between GA and CH on the inhibition of TYR was further evaluated in this study.

### 2.4. Synergistic Effect of GA and CH

Isobolographic analysis—a method for assessing whether the biological responses induced by mixtures of agents are greater, equal, or smaller than what has been expected, on the basis of the activities of individual agents and the concept of dose additivity [15]—was applied in the synergism study on GA and CH.

**Figure 5:**
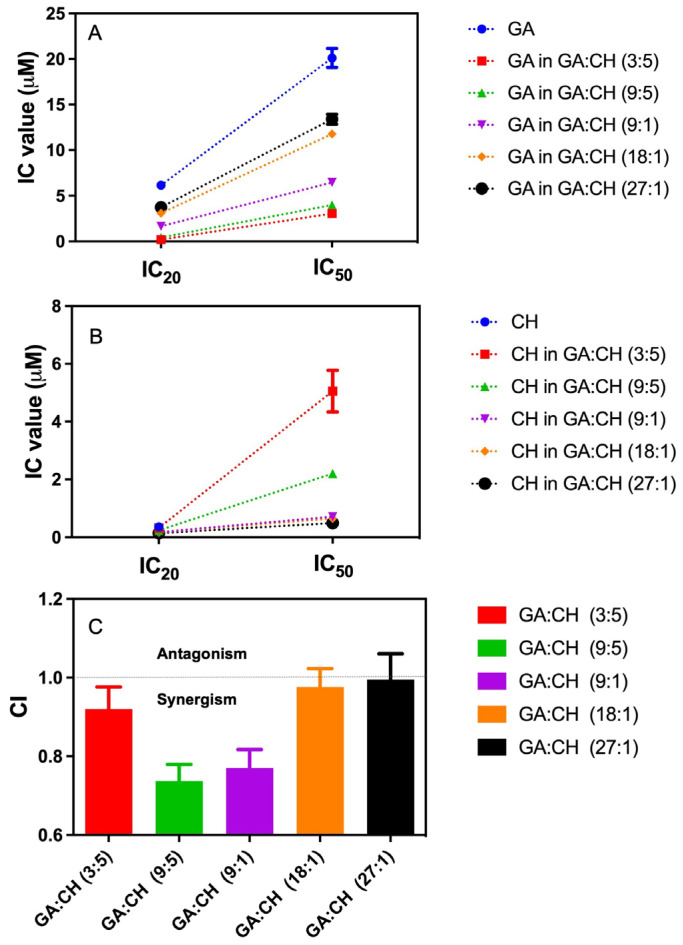
IC values of (**A**) GA and (**B**) CH in combined mixtures of different molecular ratios, and (**C**) CI value of GA:CH combinations at the inhibition level of 20% (IC_20_). GA, gallic acid; CH, catechin hydrate; IC, inhibition concentration; CI: combination index.

The 20% maximal inhibitory concentration (IC_20_) values of the GA and CH were determined for the ratio screening of GA:CH combinations. Based on the ratio of GA and CH contents in the extract (around 3:5 in molar concentration) and ratio of IC_20_ values (around 17:1), the solutions of GA and CH combinations in five ratios of 3:5, 9:5, 9:1, 18:1, and 27:1 were prepared for investigating the possible synergism. The inhibitory effects of different GA:CH combinations on tyrosinase activity are shown in Figure 5. The addition of CH in an increased ratio resulted in a proportional decrease of the IC_20_ and IC_50_ values of GA from 6.15 µM to 0.19 µM, and from 20 µM to 3.1µM, respectively (Figure 5A). Both of the IC_20_ and IC_50_ values of GA reached the minimum at a molecular ratio of GA:CH = 3:5. Meanwhile, the strongest inhibiting effect of CH alone could not reach 50% inhibition on TYR. However, with the combination of GA, CH could inhibit 50% TYR at a concentration even lower than 0.52 μM (Figure 5B), and the IC_20_ of CH on TYR was reduced from 0.36 μM to 0.14 μM. This indicated that the combination of GA and CH might enhance the inhibiting effect on TYR of each other.

To evaluate the interactive effect of GA and CH, the combination index (CI) values at IC_20_ of different GA:CH combinations were calculated and shown in Figure 5C. The CI value presents the degree of drug interaction between two or more compounds [16]. A CI near 1 indicates an additive effect, <1 synergism, and >1 antagonism of the combined compounds. According to the results, GA and CH in the ratio of 18:1 and 27:1 showed an additive effect on TYR with CI values around 1.00, and 3:5 showed an additive or weak synergistic inhibition on TYR with the CI value of 0.92. Moreover, the ratios of 9:5 and 9:1 showed a much more obvious synergism of TYR inhibition by CI < 0.77, of which 9:5 displayed the strongest synergistic effect. It suggested that certain combinations of GA and CH could increase their inhibiting activity on TYR.

## 3. Discussion

Plants are the resources of active agents against human diseases. In the last decades, several extracts generated from plants were positively screened on TYR inhibition, which could be beneficial for repairing pigmentation problems such as freckles, melasma, age spots, and acne scars [17]. However, the identification of active compounds in an extract derived from natural sources was a real challenge. These extracts might contain hundreds to thousands of compounds in varying concentrations that potentially contribute to the biological effects, to a greater or lesser extent. Moreover, the biological effects of natural extracts were often influenced by the synergism, additive action, or antagonism among compounds within them [16]. As a consequence, the traditional identification of the bioactive compound(s) in the extract by bioassay-guided fractionation often failed, since bioactivity could be lost when two or more synergists were separated from each other during the fractionation process [6]. The development and opportunities of the “omics” technologies, such as metabolomics, might be a better alternative to bioassay-guided fractionation to identify constituents relevant to the biological effect of complex mixtures [18].

Instead of traditional bioassay-guided fractionation, which is often costly and time-consuming, metabolomics analysis based on the integration of chemical profiles with biological activity profiles enables the isolation efforts to be targeted toward active rather than abundant constituents [19]. Meanwhile, it is worth mentioning that there could be possibilities of missing highly active compounds when they are present at low concentrations, and also of predicting false positives that happen to be present in the same pools and at the same relative concentrations as true active ones. That is an inherent limitation, not only of the metabolomics analysis employed here, but of any bioassay-guided fractionation experiment [20]. By utilizing optimized parameters and multiple models for data processing and acquisition, it is possible to lower the chance of falsehood. Additionally, the validation of the predicted results is always indispensable. In this study, two compounds of GA and CH were predicted to be the major active ones in RP to inhibit TYR based on OPLS analysis. To validate the prediction, the anti-TYR effects of single GA and CH were investigated. GA showed a certain degree of inhibition, as it was able to be oxidized as a substrate by the enzyme, and inhibit the synthesis of melanin through regulating the production of TYR. In contrast, the inhibiting effect of single CH was rather low. This is due to the similar structure of CH with L-tyrosine, a substrate of TYR with a phenolic group. In the presence of CH, the affinity of the enzyme for the substrate could be diminished. Catechin is the monomer unit of condensed tannins, which could not directly interact with dopachrome, but could compete by means of nucleophilic attack with the internal cyclization, thus preventing the dopachrome formation. Therefore, when L-DOPA was oxidized, CH might have already been present in the medium to interact with o-dopaquinone before the cyclization took place [21]. According to the results, the inhibition of single GA or CH was not as strong as a traditional TYR inhibitor such as KA. Thus, the reason that the two compounds were screened by the model could be due to their relatively higher distribution in the extract.

In addition, since the antagonism or synergism of multiple constituents could mask their actual activities and distort metabolomics models, we hypothesized that there might be synergy between these compounds. Synergy is assumed to occur if the effective concentration of components in combination is significantly reduced, or the effects of components in combination are significantly increased, compared to each individual component [22,23], which contributes to the widely accepted multi-drug therapy in health care. The ideal goal for using a combination of different agents with similar bioactivity is to enhance the therapeutic effect and reduce the toxicity of a single agent. For example, baicalein and daidzein, two flavonoids, could synergistically process significant neuroprotective effects against Aβ-induced cytotoxicity in PC_12_ cells [24]. However, synergy cannot be easily distinguished from additive effects, and usually relies on high margins of variation. Therefore, the isobologram analysis has been developed and performed to study the possible interactive effect among drugs [15]. In our research, it was applied to investigate the combination effect of GA and CH. According to the results, compared with the concentration of single compound, the combination of GA and CH produced the same level of the TYR inhibiting effect with much lower concentration, indicating certain combinations of them could enhance the efficacy. This finding may provide some inspirations for the development of promising natural products with TYR inhibiting activity in the future.

## 4. Materials and Methods

### 4.1. Chemicals and Reagents

KA (>98.5%), CH (>98%), L-DOPA (>98%) and mushroom TYR (≥1000 units/mg solid) were purchased from Sigma Aldrich (St. Louis, MO, USA). GA was generously provided by Natural Product Laboratory, Leiden University and its purity (>98%) was examined by ^1^H-NMR spectroscopy. As solvents used for pressurized speed extraction, *n*-hexane was purchased from the VWR Chemicals (Rosny-sous-Bois, Île-de-France, France) and acetone from Sigma-Aldrich (St. Louis, MO, USA), and all were HPLC grades.

### 4.2. RP Sample

Dried RP root and rhizome were purchased from the commercial market in Bozhou, Anhui in China, in Sep. 2017 (batch number: 20, 170, and 914) [25]. The samples were positively identified by Prof. Hongwei Wu at China Academy of Chinese Medical Sciences, Beijing. The voucher specimen is stored at Fytagoras B.V. The samples were ground with a blender and stored at room temperature.

### 4.3. PSE

PSE was performed using a Speed Extractor E-916 (BÜCHI Labortechnik AG, Flawil, Switzerland) based on the method described by Bayona et al. [12]. In total, 500 mg of dried and homogenized plant powder mixed with 9 mL fat free quartz sand (0.3–0.9 mm, BÜCHI Labortechnik AG, Flawil, Switzerland), was extracted with the solvent mixtures of n-hexane–acetone–water (Table 1) in 10 mL stainless steel extraction cells at 100 °C and 50 bar. Then, 2 cycles (heat-up time of 1 min, hold time of 1 min for cycle 1 and 2 min for cycle 2, and discharge time of 2 min) were performed.

### 4.4. NMR Analysis

Extracts were transferred to 2 mL Eppendorf tubes and dissolved in 1 mL DMSO-*d*_6_. The extracts were vortexed vigorously and sonicated for 5 min. After centrifugation at 13,000 rpm for 10 min, 300 uL of the supernatant were transferred to 3 mm-NMR tubes for ^1^H-NMR measurement. ^1^H-NMR analysis was performed using the method described by López-Gresa et al. [26]. Briefly, ^1^H-NMR spectra were recorded at 25 °C on a 600 MHz Bruker AV 600 spectrometer equipped with a TCI cryo-probe. DMSO-*d*_6_ was used for the internal lock. Each ^1^H-NMR spectrum consisted of 64 scans requiring 5 min acquisition time. The experimental parameters were as follows: 0.25 Hz/point, pulse width = 30° (10.8 ms), and relaxation delay = 1.5 s. Free induction decays were Fourier transformed with Line Broadening = 0.3 Hz and the spectra were zero-filled to 32 K points. The resulting spectra were manually phased, and baseline corrected, and calibrated to DMSO at 2.49 ppm using Topspin (version 3.5, Bruker).

### 4.5. Data Processing and Multivariate Data Analysis

For the multivariate data analysis, ^1^H-NMR spectra were imported to an Excel file using AMIX 3.9.12 (Bruker, Germany). Bucket data were obtained by spectra integration at every 0.04 ppm from δ 10.02 to 0.20. The peak intensity of individual peak was scaled to the total intensity of the peaks. The regions δ 3.36–3.24 and δ 2.44–2.54 were excluded from the analysis because of the signal of residual solvent and DMSO-*d*_6_, respectively. The OPLS method was performed with the SIMCA-P software (v. 16.0.2, Umetrics, Umeå, Sweden). We used the unit variance scaling method for OPLS modelling. ^1^H-NMR signal identification was performed using in-house database and/or comparison with published reference data.

### 4.6. TYR Inhibition Assay

The dry extracts/compounds were dissolved in 50% DMSO to prepare sample solutions of different concentrations. The 5 mM L-DOPA and 200 U/mL TYR solutions were prepared by dissolving each of them in 67 mM phosphate buffer solution (PBS, pH 6.8), respectively. The inhibition of TYR was determined using the modified dopachrome method with L-DOPA as the substrate [27]. The spectrophotometric assays were performed in 96-well microplate using an ELISA microplate reader. A total of 10 µL of sample was added to 96-well micro plate. Then, 80 µL of 67 mM phosphate buffer (pH 6.8) and 30 µL of 5 mM L-DOPA were added in each well. After incubating at 37 °C for 10 min, 30 µL of TYR solution (200 U/mL) was added. The reaction was monitored after 20 min for the formation of dopachrome by measuring the optical absorbance at 492 nm using the microplate reader. All experiments were carried out in triplicates. Results were compared with the control (50% DMSO). KA was used as the positive control. The inhibition rate of TYR (*I*%) was calculated according to the Equation (1):

*I* % = [(*C* − *C*_0_) − (*A* − *A*_0_)]/(*C* − *C*_0_) × 100 (1)

where *C* is the absorbance of TYR reacting with L-DOPA, *C*_0_ is the absorbance of PBS and L-DOPA, *A* is the absorbance of TYR reacting with L-DOPA after adding the sample, and *A*_0_ is the absorbance of PBS and L-DOPA after adding the sample.

### 4.7. Synergistic Effect of GA and CH

The synergistic effect of GA and CH was investigated by isobologram analysis, which provided a CI value to present the degree of drug interaction between two or more compounds [16]. The CI value was calculated by a method described by Chou and Talalay [28] and Chou [22] according to the Equation (2):

(2) CInx=∑j=1nDj/Dxj=Dx1−nDj∑j=1nDDmjfaxj/1−faxj1/mj

where CInx is the CI for *n* compounds (e.g., GA and CH) at *x*% inhibition (e.g., inhibition on tyrosinase); ${\left(D_{x}\right)}_{1-n}$ is the sum of the concentration of n compounds that exerts *x*% inhibition in combination; $\left\{\left[D_{j}\right]/\sum_{j=1}^{n}\left[D\right]\right\}$ is the proportionality of the concentration of each of n compounds that exerts *x*% inhibition in combination; and ${\left(D_{m}\right)}_{j}{\left\{{\left(fax\right)}_{j}/\left[1-{\left(fax\right)}_{j}\right]\right\}}^{1/mj}$ is the concentration of each compound alone that exerts *x*% inhibition. A CI near 1 indicates an additive effect, <1 synergism, and >1 antagonism of the combined compounds.

### 4.8. Statistical Analyses

All data are expressed as means ± standard deviation. The isobologram analysis and one way-ANOVA were performed using GraphPad Prism 6 (GraphPad, San Diego, CA, USA). The OPLS method was performed with the SIMCA-P software (v. 16.0.2, Umetrics, Umeå, Sweden). A value of *p* < 0.05 was considered significant; values of *p* < 0.01 and *p* < 0.001 were considered highly significant.

## 5. Conclusions

In the present study, the TYR inhibition effect of RP was examined. By using a metabolomics approach, the corresponding active components of RP were identified to be GA and CH. To better elucidate and improve the anti-TYR activity of the two active compounds, their synergism was studied. The results showed that GA and CH, in the ratios of 9:5 and 9:1, exhibited a synergistic inhibition on TYR, suggesting that certain combinations of RP compounds could be effective for use in cosmetic, pharmaceutical, and food industries. Meanwhile, additional works are required to clarify the possible mechanism and toxicity of related products.

## Figures and Tables

**Table 1:** Solvent composition used for the speed extraction.

	Solvents Composition (%)		
Extracts	*n*-Hexane	Acetone	Water
RPE1	100	0	0
RPE2	65	35	0
RPE3	35	65	0
RPE4	0	100	0
RPE5	0	65	35
RPE6	0	35	65
RPE7	0	0	100

## Data Availability

The data presented in this study are available on request from the corresponding author.

## Supplementary Materials

The following supporting information can be downloaded at: https://www.mdpi.com/article/10.3390/molecules28030944/s1, Figure S1: Figure S1. ^1^H-NMR spectra of *Rheum palmatum* extract (RPE4) in DMSO-*d*_6_.

## Sample Availability

Samples of the compounds are not available from the authors.

## Abbreviations

CH	catechin hydrate
CI	combination index
DMSO	dimethyl sulfoxide
GA	gallic acid
IC	inhibitory concentration
KA	kojic acid
L-DOPA	3,4-dihydroxy-L-phenylalanine
NMR	nuclear magnetic resonance
OPLS	orthogonal partial least squares to latent structures
PSE	pressurized solvent extraction
RP	*Rheum palmatum*
RPE	*Rheum palmatum* extract
TYR	tyrosinase
