# Supplementary material for: Synergistic Inhibiting Effect of Phytochemicals in Rheum palmatum on Tyrosinase Based on Metabolomics and Isobologram Analyses
Source: Molecules. 2023 Jan 17;28(3):944. doi: 10.3390/molecules28030944 (PMC9919157; doi:10.3390/molecules28030944)
Supplement: Supplementary file 1 [file molecules-28-00944-s001.zip › molecules-2131025-supplementary.pdf]

Supplement materials

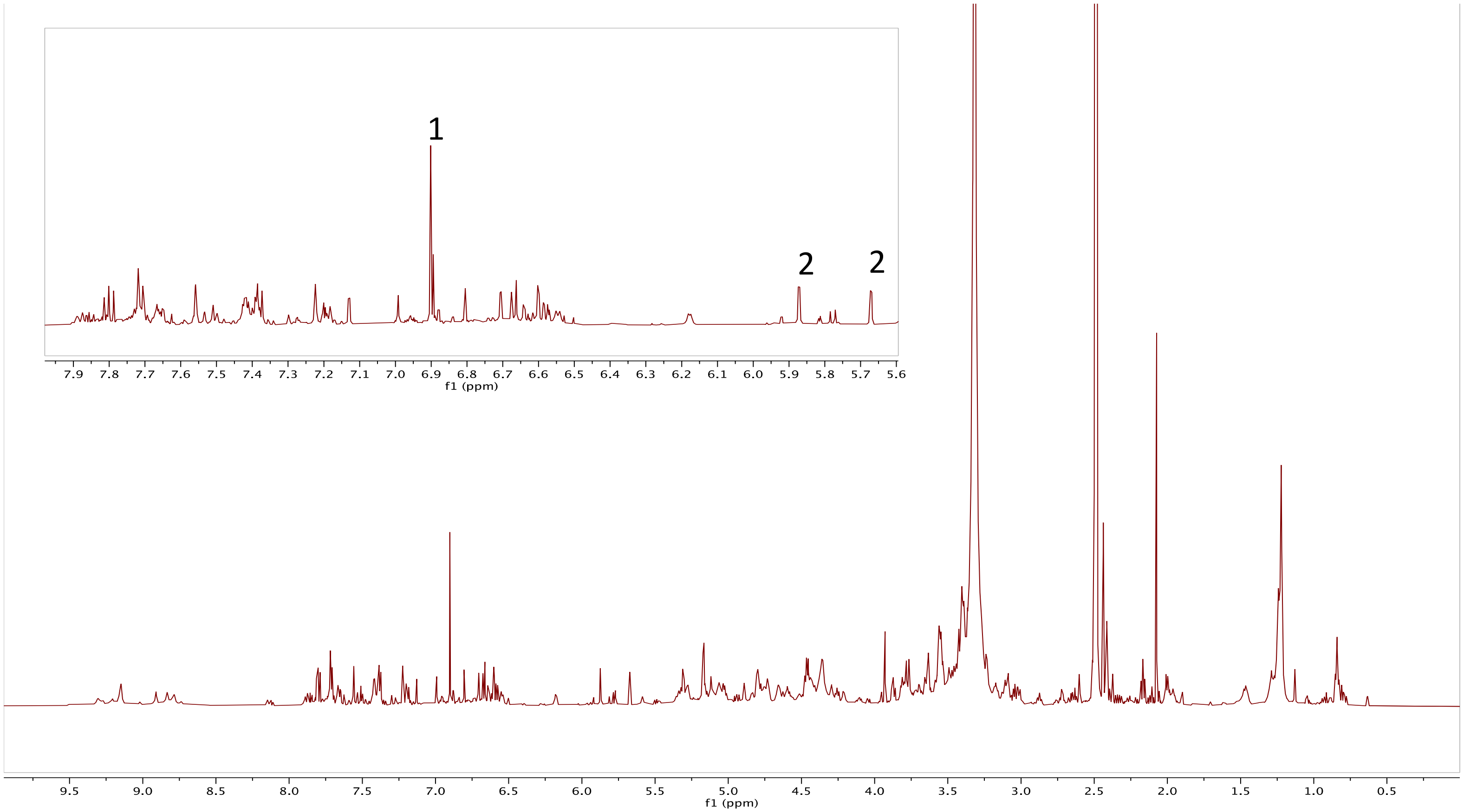

Figure S1. ^{1}H-NMR spectra of *Rheum palmatum* extract (RPE4) in DMSO-d_6. The spectrum of all range is shown and the expansion of aromatic area is inserted on top. 1, signal of gallic acid (δ 6.90 [s]); 2, signals of catechin (δ 5.87 [d, J= 2.4 Hz], and δ 5.67 [d, J=2.4 Hz]).
